# Learning and interpreting the gene regulatory grammar in a deep learning framework

**DOI:** 10.1371/journal.pcbi.1008334

**Published:** 2020-11-02

**Authors:** Ling Chen, John A. Capra

**Affiliations:** 1 Department of Biological Sciences, Vanderbilt University, Nashville, TN, United States of America; 2 Vanderbilt Genetics Institute and Department of Biomedical Informatics, Vanderbilt University Medical Center, Nashville, TN, United States of America; 3 Department of Computer Science, Vanderbilt University, Nashville, TN, United States of America; University of Wisconsin, Madison, UNITED STATES

## Abstract

Deep neural networks (DNNs) have achieved state-of-the-art performance in identifying gene regulatory sequences, but they have provided limited insight into the biology of regulatory elements due to the difficulty of interpreting the complex features they learn. Several models of how combinatorial binding of transcription factors, i.e. the regulatory grammar, drives enhancer activity have been proposed, ranging from the flexible TF billboard model to the stringent enhanceosome model. However, there is limited knowledge of the prevalence of these (or other) sequence architectures across enhancers. Here we perform several hypothesis-driven analyses to explore the ability of DNNs to learn the regulatory grammar of enhancers. We created synthetic datasets based on existing hypotheses about combinatorial transcription factor binding site (TFBS) patterns, including homotypic clusters, heterotypic clusters, and enhanceosomes, from real TF binding motifs from diverse TF families. We then trained deep residual neural networks (ResNets) to model the sequences under a range of scenarios that reflect real-world multi-label regulatory sequence prediction tasks. We developed a gradient-based unsupervised clustering method to extract the patterns learned by the ResNet models. We demonstrated that simulated regulatory grammars are best learned in the penultimate layer of the ResNets, and the proposed method can accurately retrieve the regulatory grammar even when there is heterogeneity in the enhancer categories and a large fraction of TFBS outside of the regulatory grammar. However, we also identify common scenarios where ResNets fail to learn simulated regulatory grammars. Finally, we applied the proposed method to mouse developmental enhancers and were able to identify the components of a known heterotypic TF cluster. Our results provide a framework for interpreting the regulatory rules learned by ResNets, and they demonstrate that the ability and efficiency of ResNets in learning the regulatory grammar depends on the nature of the prediction task.

## Author summary

Gene regulatory sequences function through the combinatorial binding of transcription factors (TFs). However, the specific binding combinations and patterns that specify regulatory activity in different cellular contexts (“regulatory grammars”) are poorly understood. Deep neural networks (DNNs) have achieved state-of-the-art performance in identifying regulatory DNA sequences active in different contexts, but they have provided only limited biological insight due to the complexity of the statistical patterns they learn. In this study, we explore the power and limitations of DNNs in learning regulatory grammars through biologically motivated simulations. We simulated regulatory sequences based on existing hypotheses about the structure of possible regulatory grammars and trained DNNs to model these sequences under a range of scenarios that reflect real-world regulatory sequence prediction tasks. We developed an unsupervised clustering method to extract learned patterns from the trained DNNs and compare them to the simulated grammars. We found that our method can successfully extract regulatory grammars when they are learned by the DNN, but the ability of DNNs to learn regulatory grammars highly depends on the nature of the prediction task. Finally, we show that our DNN approach highlights a known heart regulatory grammar when applied to real mouse enhancer sequences.

## Introduction

Enhancers are genomic regions distal to promoters that regulate the dynamic spatiotemporal patterns of gene expression required for the proper differentiation and development of multi-cellular organisms [1–3]. As a result of their essential role, mutations that disrupt proper enhancer activity can lead to diseases. Indeed, the majority of genetic variants associated with complex disease in genome-wide association studies (GWAS) are non-protein coding, and thought to influence disease by disrupting proper gene expression levels [4–6].

Enhancers function through the coordinated binding of transcription factors (TFs). Recent advances in high-throughput sequencing techniques have greatly deepened our knowledge of TF binding specifies [7–9]. However, identifying consensus TF binding motifs is not sufficient for inferring TF binding. As shown in many ChIP-seq studies, TFs only bind to a small fraction of all motif occurrences in the genome, and some binding sites do not contain the consensus TF binding motif, indicating a necessity for additional features [10]. Indeed, many additional features have been suggested to play a role in determining *in vivo* TF binding, such as heterogeneity of a TF’s binding motif [11], local DNA properties [12], broader sequence context and interposition dependence [13], cooperative binding of the TF with its partners [14–17], and condition-specific chromatin context [15, 18, 19]. While both genomic and epigenomic features are important in determining the *in vivo* occupancy of a TF, recent studies have suggested that the epigenome can be accurately predicted from genomic context [12, 20–22], supporting the fundamental role of sequence in dictating the binding of TFs [23–27]. Therefore, it is critical to understand the sequence patterns underlying enhancer regulatory functions and build sufficiently sophisticated models of enhancer sequence architecture.

Combinatorial binding of TFs, i.e., the regulatory “grammar” that combines TF “words”, is thought to be essential in determining *in vivo* condition-specific binding [11, 13, 20, 28]. However, how enhancers integrate multiple TF inputs to direct precise patterns of gene expression is not well understood. Most enhancers likely fall on a spectrum represented by two extreme models of enhancer architecture: the *enhanceosome model* and the *billboard model* [29, 30]. The enhanceosome model proposes that enhancer activity is dependent on the cooperative assembly of a set of TFs at enhancers. The cooperative assembly of an enhanceosome is based on physical protein-protein interactions and highly constrained patterns of TF-DNA binding sites. The enhanceosome model does not tolerate shifts in the spacing, orientation, or ordering of the binding sites, which can disrupt protein-protein interactions and cooperativity. This model likely presents an extreme example because only very few enhancers are found under such stringent constraints [31–35]. However, many examples of less extreme spatial constraints on paired TF-TF co-association and binding-site combinations are found in genome-wide ChIP sequencing studies [36–38] and *in vitro* consecutive affinity-purification systematic evolution of ligands by exponential enrichment (CAP-SELEX) studies. On the other end of the spectrum is the billboard model, also known as the information display model [39, 40], which hypothesizes that instead of functioning as a cooperative unit, enhancers work as an ensemble of separate elements that independently affect gene expression. That is, the positioning of binding sites within an enhancer is not subject to strict spacing, orientation, or ordering rules. The TFs at billboard enhancers work together to direct precise patterns of gene expression, but their function does not strongly depend on each other. For instance, the loss of a TF binding may lead to change in the target gene expression, but will not cause the complete collapse of enhancer function. The actual mechanisms by which multiple TFs assemble on enhancers are likely a mixture of the two models. Indeed, a massively parallel reporter assay (MPRA) of synthetic regulatory sequences suggested that while certain transcription factors act as direct drivers of gene expression in homotypic clusters of binding sites, independent of spacing between sites, others function only synergistically [41].

In recent years, deep neural networks (DNNs) have achieved state-of-art prediction accuracies for many tasks in regulatory genomics, such as predicting splicing activity [42, 43], specificities of DNA- and RNA-binding proteins [44], transcription factor binding sites (TFBS) [45–47], epigenetic marks [45, 46, 48, 49], enhancer activity [50, 51] and enhancer-promoter interactions [52]. However, in spite of their superior performance, little biological knowledge or mechanistic understanding has been gained from DNN models. In computer vision, the interpretation of DNNs trained on image classification tasks demonstrate that high-level neurons often learn increasingly complex patterns building on those learned by lower level neurons [53–59]. DNNs trained on DNA sequences might behave similarly, with neurons in low levels learning building blocks of the regulatory grammar, short TF motifs, and those in higher levels learning the regulatory grammar itself, the combinatorial binding rules of TFs [46, 48, 60].

The majority of DNNs trained with genomic sequences use a convolution layer as a first layer and then stack convolution or recurrent layers on top. A common approach to interpret the features learned by such DNNs is to convert the first convolution layer neurons to position weight matrices by counting nucleotide occurrences in the set of input sequences that activate the neurons [44, 48, 60]. With the development of more advanced DNN visualization and interpretation techniques in computer vision, many other DNN interpretation methods emerged, such as occlusion [55], saliency maps [61], guided propagation [55], gradient ascent [57]. Some of these techniques have been applied to visualize features learned by DNNs trained with genomic sequences. For instance, a gradient-based approach, DeepLIFT, identified relevant transcription factor motifs in the input sequences learned by a convolutional neural network [56]. Saliency maps, gradient ascent and temporal output scores have been used to visualize the sequence features learned by a DNN model for TFBS classification and found informative TF motifs [62]. These studies demonstrate the power of DNNs in recognizing the TF motifs in the input sequences. However, these studies focused only on the interpretation of the output layer in models for predicting TFBS. Enhancers can be much more complex than individual TFBS; they contain multiple binding sites in range of combinations and organizations. It is also unclear whether the intermediate layers of DNNs have the capability of learning increasingly complex rules of combinatorial TF binding from regulatory regions with many TFs, such as enhancers.

Another substantial challenge in the development of methods to interpret DNNs applied to regulatory sequences is our lack of knowledge of the combinatorial rules governing enhancer function in different cell types. Beyond a few foundational examples used to propose possible enhancer architectures, the constraints and interactions that drive enhancer function are largely unknown. Thus, it is difficult to determine if a pattern learned by a neuron is “correct” or biologically relevant. The generation of synthetic DNA sequences that reflect different constraints on regulatory element function has promise to help address these challenges and enable evaluation of the ability of DNNs to learn different regulatory architectures and of algorithms for reconstructing these patterns from the trained networks. Indeed, DeepResolve was recently proposed to interpret the combinatorial logic from intermediate layers of DNNs, and the ability of the neural network to learn the AND, OR, NOT and XOR of two short sequence patterns was demonstrated in a synthetic dataset [63]. However, these simulated combinatorial logics and sequence patterns were not biologically motivated and were simpler than most proposed enhancer architectures.

Here, we develop a biologically motivated framework for simulating enhancer sequences with different regulatory architectures, including homotypic clusters, heterotypic clusters, and enhanceosomes, based on real TF motifs from diverse TF families. We then apply a state-of-the-art variant of deep neural networks, residual neural network (ResNet) algorithms, to classify these sequences. Compared to previous DNNs, ResNets have “skip” connections between layers that enable the training of deeper network architectures We chose ResNets over other DNNs, because of their deeper structures and state-of-the-art performance in computer vision. We use this framework to investigate whether the intermediate layers the networks learn the complex combinatorial TF architectures present in the simulated regulatory grammars. In particular, we developed an unsupervised method for assigning transcription factor binding sites to grammars based on the gradients assigned to their nucleotides by intermediate layers of the neural network. We evaluate the efficiency in extracting simulated regulatory grammars under a range of scenarios that mimic real-world multi-label regulatory sequence prediction tasks, considering possible heterogeneity in the output enhancer categories and fraction of TFBS not in the regulatory grammar. We demonstrate that ResNets can accurately model simulated regulatory grammars in many multi-label enhancer prediction tasks, even when there is heterogeneity in the output categories or a large fraction of TFBS outside of regulatory grammar. We also identified scenarios where the ResNet fails to learn an accurate representation of the regulatory grammar, including using inappropriate sequences as negative training examples, considering output categories differing in multiple sequence features, and having an overwhelming amount of TFBS outside of the regulatory grammar. Finally, we trained a ResNet on mouse developmental enhancer sequences from 12 tissues and demonstrated that it identifies and clusters the binding sites of the known heart heterotypic cluster consisting of TBX5, NKX2-5, and GATA4 [64].

In summary, our work makes three main contributions: i) We provide a flexible tool for simulating regulatory sequences based on biologically driven hypotheses about regulatory grammars. ii) We develop and evaluate an algorithm for interpreting the regulatory grammar from the intermediate layers of DNNs trained on enhancer DNA sequences. iii) We demonstrate that the ability of DNNs to learn interpretable regulatory grammars is highly dependent on the design of the prediction task.

## Results

**Fig 1:**
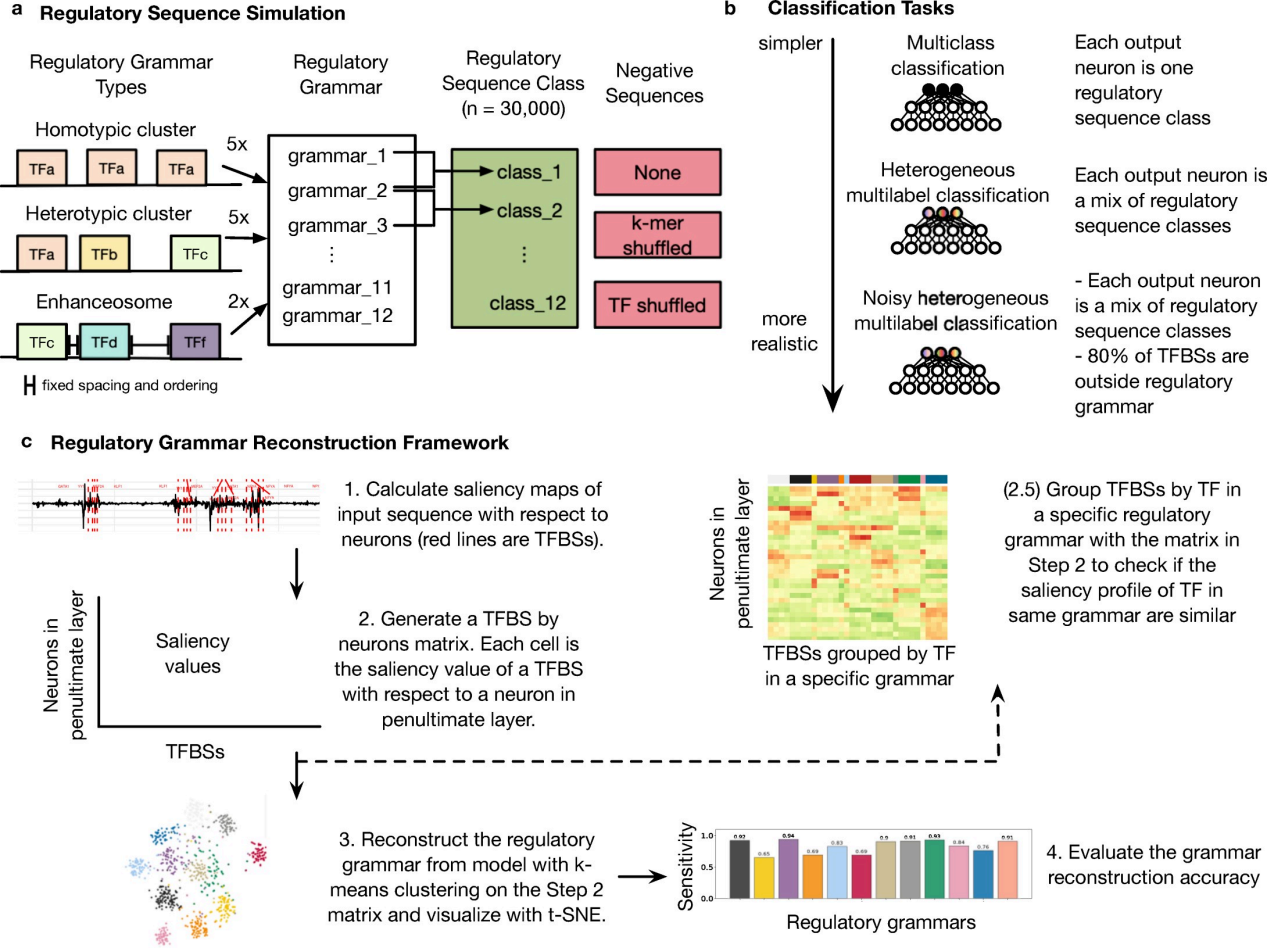
Pipeline for analyzing regulatory grammar learned by ResNet models trained on simulated regulatory sequences. (a) Regulatory sequence and negative sequence simulation. We designed twelve regulatory grammars, including five homotypic clusters, five heterotypic clusters, and two enhanceosomes as prototypes for simulated regulatory sequences. Then, to reflect that regulatory regions active in a cellular context may have multiple grammars, we defined twelve regulatory sequence classes, each with two different grammars. Finally, we generated two sets of negative sequences: k-mer shuffled and TF shuffled versions of the simulated positive sequences. (b) Classification tasks. ResNets are trained on simulated regulatory sequences and the negative sets in three increasingly realistic scenarios. (c) Regulatory grammar reconstruction framework.

A common task in regulatory sequence analysis is to predict enhancers’ activity in different cellular contexts. Enhancers active in different cellular contexts may harbor unique sets of context-specific TFBSs as well as similar sets of binding sites for broadly active transcription factors. To evaluate the performance of ResNets on modeling the regulatory grammar, we performed a series of simulation analyses (Fig 1), which we designed with increasing complexity that mimics challenges faced in analysis of real enhancers.

We first designed a set of 12 biologically motivated regulatory grammars consisting of TFs from diverse families (Fig 1A). These include five homotypic clusters of the same TF, five heterotypic clusters of different TFs, and two enhanceosomes of different TFs with requirements on the spacing and orientation of their binding sites. Motivated by the fact that enhancers active in a given cellular context likely consist of multiple types with different grammars, we designed twelve “classes” of regulatory sequences. Each class contains a different set of regulatory grammars, but the grammars can occur within multiple classes, and TFs can occur within multiple grammars. The classes can be thought of as representing the different enhancers active in a specific cellular context. Then, using these classes, we simulated 30,000 enhancer sequences, which each contain a sequence that matches the pattern defined by one of the classes (Methods). We also simulated three sets of non-enhancer sequences to evaluate how the choice of negatives has an impact on what the model can learn from the data. The three negative sets are: 1) no negatives, 2) k-mer matched negatives, and 3) TF-shuffled negatives. We generated k-mer matched negatives with k = 1, 2, 4, 8, 12. We generated TF-shuffled negatives by randomly switching the positions of TFBSs in the simulated enhancers to break the link between a TFBS and its associated regulatory grammar.

Next, we designed three increasingly complex scenarios for modeling enhancers based on real-world regulatory sequence prediction tasks using the sequences generated from the simulated regulatory grammars (Fig 1B). The first scenario is multi-class classification where each output neuron corresponds to one regulatory class. The second scenario is heterogenous multilabel classification where each output neuron corresponds to a mix of sequences from different regulatory classes. We designed this scenario because it is likely in real enhancer analysis. Enhancers active in one tissue may represent different cell types or cell states present in the sample, and thus belong to different regulatory classes. The third scenario is noisy heterogenous multilabel classification where we added TF binding sites that are not in any grammar into the simulation. This reflects that it is likely that the majority of TF binding sites in an enhancer are not in any regulatory grammar.

We then trained ResNet models for each of these scenarios against three choices of negatives. Finally, we interpreted the grammar learned by the model using a saliency-map-based method for TF binding site clustering and compared the ability of the ResNet to learn simulated regulatory grammars under each scenario.

### ResNet trained on simulated regulatory sequences and TF-shuffled negatives accurately captures simulated regulatory grammars

To explore whether the ResNet model can learn the regulatory grammar, we started with a multi-class classification task based on simulated regulatory sequences from 12 classes and TF-shuffled negative sequences (Methods; S1 Table and S2 Tables). We trained a classifier to predict the class of the sequence, either not a regulatory sequence or member of one of the regulatory sequence classes. By constructing the prediction task with TF matched negative sequences, the neural network is forced not only to learn the individual TF motifs, but also learn the combinatorial patterns between the TFs.

**Fig 2:**
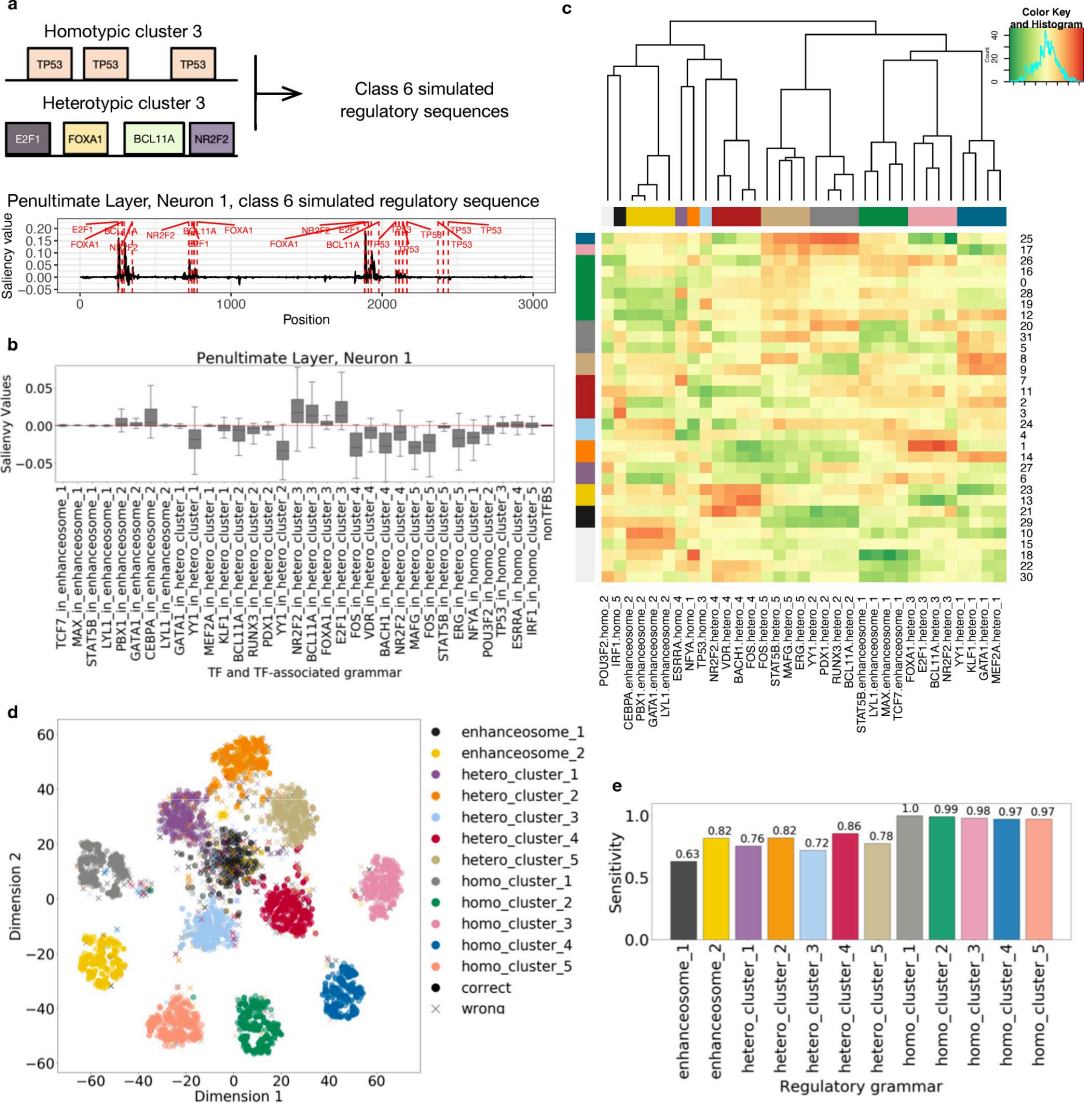
ResNet trained on simulated regulatory sequences and TF-shuffled negatives accurately models the regulatory grammar. (a) Example saliency map for a simulated regulatory sequence from class 6. Class 6 sequences harbor instances of homotypic cluster 3 and heterotypic cluster 3. The saliency map shown is computed with respect to neuron 1 in the penultimate layer. The red dashed lines show simulated TFBSs in their respective regulatory grammars. (b) The saliency values of the binding sites of each TF in a specific regulatory grammar with respect to neuron 1 in the penultimate layer. (c) Heatmap of the median saliency value of the binding sites of each TF in a specific regulatory grammar (x axis) across neurons of the penultimate layer (y axis). The order of x and y axis labels are determined by hierarchical clustering. The color bars on the side indicate the group label assigned by hierarchical clustering. (d) Actual labels of simulated regulatory grammar of the TFBS overlaid on t-SNE visualization of TFBS saliency values across neurons. Correct prediction of the regulatory grammar for a TF (the predicted label agrees with the actual label) is represented by a dot. Incorrect prediction of the regulatory grammar of a TF is indicated by an “x”. (e) The sensitivity (TP/(TP+FN)) of the regulatory grammar predictions.

The ResNet model accurately predicts the class label of input DNA sequences with near perfect performance: average area under the ROC curve (auROC) of 0.999 and average area under the precision-recall curve (auPR) of 0.982. We then analyzed what features were learned by calculating saliency maps (Methods) of input sequences with respect to each neuron in the penultimate layer (the dense layer immediately before the output layer). We found that neurons in the penultimate layer detect the location of the simulated TFBS. For instance, when we compute the saliency map of a class 6 simulated regulatory sequence with respect to neuron 1 in the penultimate layer, the TFBSs have higher absolute saliency value compared to other locations in the sequence, indicating the higher importance of those nucleotides to the activation of neuron 1 (Fig 2A and 2B).

Next, we visualized the features learned by neuron 1 of the penultimate layer by plotting the mean saliency value of a 10 bp window from the start of each TF binding site using 240 sequences from all simulated regulatory sequence classes (Fig 2B). For example, the TFBSs from heterotypic cluster 3 have elevated gradients compared to TFBSs from other simulated regulatory grammars. This suggests that neuron 1 of the penultimate layer detects TFBSs from heterotypic cluster 3. We then took the median saliency values of TFBSs in a specific regulatory grammar and generated a matrix with rows of neurons and columns of each TF. As shown in Fig 2B, although TFBSs from heterotypic cluster 3 have elevated saliency values, those values are not always at the same level. For example, FOXA1 binding sites have lower median saliency value than the other three TFs in the grammar. Therefore, we scaled the matrix column-wise to identify which TF is most learned by which neuron. We plotted the scaled matrix as a heatmap with hierarchical clustering (Method; Fig 2C). We found that: (i) TFBSs from the same regulatory grammar have elevated gradients together and therefore are clustered; (ii) neurons of the penultimate layer can “multi-task”, that is, one neuron can detect one or more regulatory grammars. For instance, neuron 25 in the penultimate layer learned both heterotypic cluster 2 and 5. This suggests that the penultimate layer captured the simulated regulatory grammars.

In the above analyses, we used the simulation information to group TFBSs by their TF motifs and regulatory grammar and demonstrated that neuron activation patterns for each regulatory grammar are different. However, in real enhancer analysis, we do not have access to this information. Therefore, we need to evaluate whether we can reconstruct the regulatory grammar solely based on the saliency values of TFBSs. To test this, we performed unsupervised clustering of TFBSs based on their saliency values with respect to the neurons in the penultimate layer. More specifically, we performed a k-means clustering (k = 12) of TFBSs from 240 sequences using their saliency values with respect to each neuron of the penultimate layer and visualized it with t-SNE (Fig 2D). Each TFBS has a predicted clustering label that is assigned by the k-means clustering algorithm and a true regulatory grammar. We used majority voting to determine the predicted regulatory grammar for a cluster. For instance, the majority of cluster 1 is from heterotypic cluster 1, so we assign heterotypic cluster 1 as the predicted regulatory grammar for all TFBS in cluster 1. We then calculate the accuracy and sensitivity of the regulatory grammar reconstruction by comparing the predicted regulatory grammar and the true regulatory grammar. On average, 85.1% of TFBS are correctly classified (Fig 2E), and homotypic clusters are generally learned better (sensitivity > 0.97) than heterotypic clusters and enhanceosomes.

The same analysis approach can be applied to any layer of the neural network. We found that the neural network built up its representation of the regulatory grammar by first learning the individual TF motifs in the lower level neurons and gradually grouping TF motifs in the same regulatory grammar together (S3 Fig).

Taken together, these results demonstrate that ResNet models can largely capture simulated regulatory grammars if trained to perform a multi-class prediction with TF-shuffled negatives, and that our unsupervised clustering method based on saliency maps is able to reconstruct the regulatory grammar.

### Regulatory grammar can be learned by the ResNet model without TF-shuffled negatives

Although the ResNet model demonstrated the ability to capture the simulated regulatory grammars when trained against TF-shuffled negatives, we cannot construct perfect TF-shuffled negatives in the real-world, because the true TFs are not known. Indeed, in many applications, only the positive regulatory sequences [45, 46, 65] or k-mer shuffled negatives are used for training machine learning models. Therefore, we tested whether the ResNet model can learn the simulated regulatory grammar if trained with no negatives or k-mer shuffled negatives.

**Fig 3:**
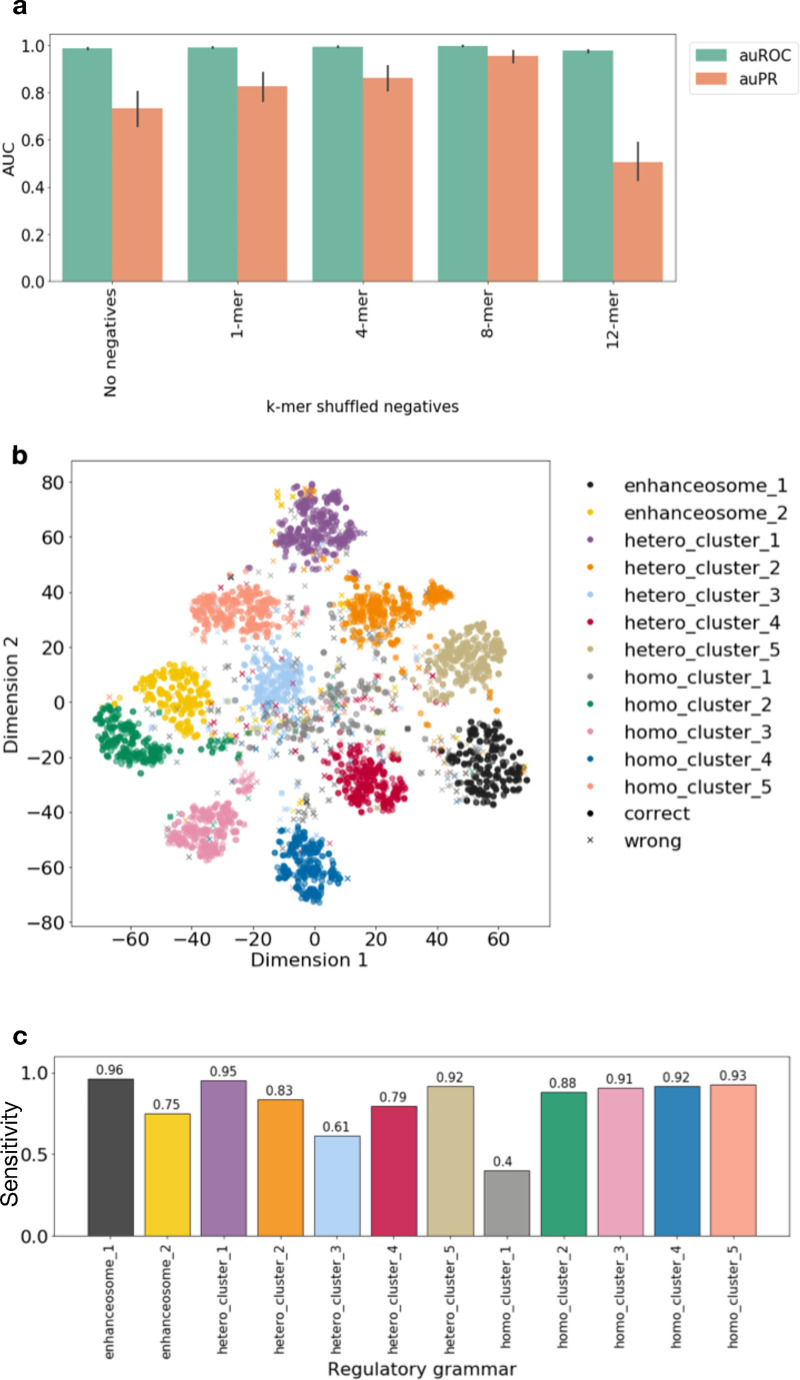
ResNet trained on simulated regulatory sequences against 8-mer shuffled negatives accurately models the regulatory grammar. (a) The performance of five different ResNet models trained on simulated regulatory sequences against different k-mer shuffled negatives at predicting the regulatory class of the simulated regulatory sequences vs. TFs-shuffled negatives test dataset. (b) Actual labels of simulated regulatory grammar of the TFBS overlaid on t-SNE visualization of TFBS saliency values across neurons. (c) The sensitivity of predicted labels in (b) of the ResNet model trained on the simulated regulatory sequences against 8-mer shuffled negatives.

We trained five models for multi-class classification against: no negatives, 1-mer shuffled negatives, 4-mer shuffled negatives, 8-mer shuffled negatives, and 12-mer shuffled negatives. Then, we evaluate their performance at predicting simulated regulatory sequences. The model trained with 8-mer shuffled negatives achieved the highest accuracy at distinguishing TF-shuffled negatives from simulated regulatory sequences (average auROC 0.998, auPR 0.957, Fig 3A).

To further explore the regulatory grammar learned by the ResNet model trained against 8-mer shuffled negatives, we calculated saliency maps over a set of input sequences (n = 240) from each class of simulated regulatory sequences with respect to neurons in the penultimate layer. We performed hierarchical clustering on the median saliency values for the binding sites for each TF in a specific regulatory grammar as we did in the previous results section (S4 Fig). We found that TFBS from the same regulatory grammar were grouped together. Next, we performed k-means clustering (k = 12) of the TFBS from the 240 sequences and overlaid the clustering label on the tSNE visualization (Fig 3B). We calculated the accuracy of predicted regulatory grammar for each TF. The average grammar reconstruction accuracy of this model is on par with the model trained against TF-shuffled negatives (85.3% vs. 85.1%).

These results suggest that the model trained against 8-mer shuffled negatives can learn a good representation of the regulatory grammar and therefore 8-mer shuffled negatives can be used as a substitute for TF-shuffled negatives in practice.

### Regulatory grammar can be learned by the ResNet model in the presence of heterogeneity in the regulatory sequences

A common task in regulatory sequence prediction is to identify sequences with a certain set of functions, e.g., activity in different cellular contexts. However, it is likely that sequences with a heterogeneous set of many grammars are active in each cellular context.

To mimic this type of heterogeneity, we performed a heterogenous multi-label classification by pooling a number of simulated regulatory classes together as one heterogeneous class to generate five heterogeneous classes (Methods; Fig 1B, S4 Table). We also allowed one regulatory class to be used in several heterogeneous classes. For example, in our simulation, regulatory sequences in heterogenous class 1 consist of regulatory class 1, 3, and 5. Regulatory class 1 sequences also belong to heterogenous class 5, and regulatory class 5 sequences also belong to heterogenous class 4. This multi-function of a regulatory sequence class is often observed in real-word regulatory sequences as many enhancers are active in more than one cellular context.

**Fig 4:**
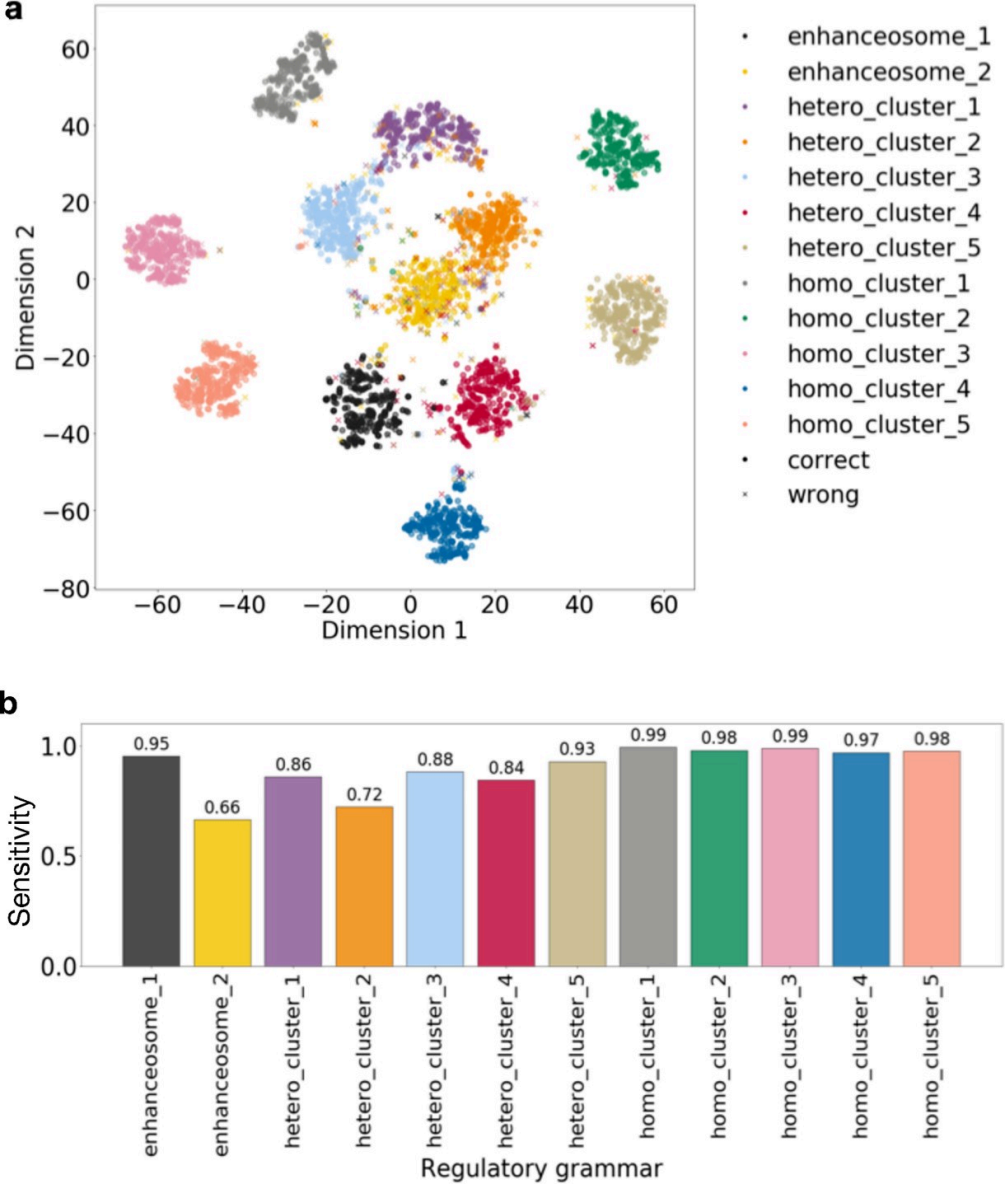
Regulatory grammar can be learned by ResNet despite heterogeneity in the regulatory sequences. (a) Actual labels of simulated regulatory grammar of the TFBS overlaid on t-SNE visualization of TFBS saliency values across neurons. (b) The sensitivity of predicted labels in (a) across regulatory grammars.

We trained the ResNet model against k-mer shuffled negatives (k = 1, 4, 8, 12). Again, the model trained against 8-mer shuffled negatives performed the best when evaluated against the TF-shuffled negatives (average auROC 0.99, auPR 0.93, S5A Fig). We performed hierarchical clustering (S5B Fig) and unsupervised clustering (Fig 4A and 4B) as we did in the previous sections. The model trained to predict the heterogenous classes can still learn the majority of the regulatory grammars. The average accuracy of reconstructing regulatory grammar in this setting is 89.2%, which is similar to that of the multi-class classifications against TF-shuffled negatives (85.1%) and against k-mer shuffled negatives (85.3%).

These results suggest that the model trained on regulatory sequences with heterogenous output categories can still largely capture the regulatory grammars that are essential for the heterogenous multi-label classification.

### Regulatory grammar can be learned by ResNet when a large fraction of TFBSs are not in grammars and there is heterogeneity in the regulatory sequences

In all previous prediction tasks, the simulated TFBSs in the input sequences are always in a regulatory grammar. However, in real regulatory sequences, it is likely that only a fraction of TFBS are in regulatory grammars, while others are individual motifs scattered along the sequence. To mimic this scenario, we simulated a set of regulatory sequences with 80% of TFBSs randomly scattered in the sequence outside of any regulatory grammar and 20% of TFBSs in regulatory grammar.

**Fig 5:**
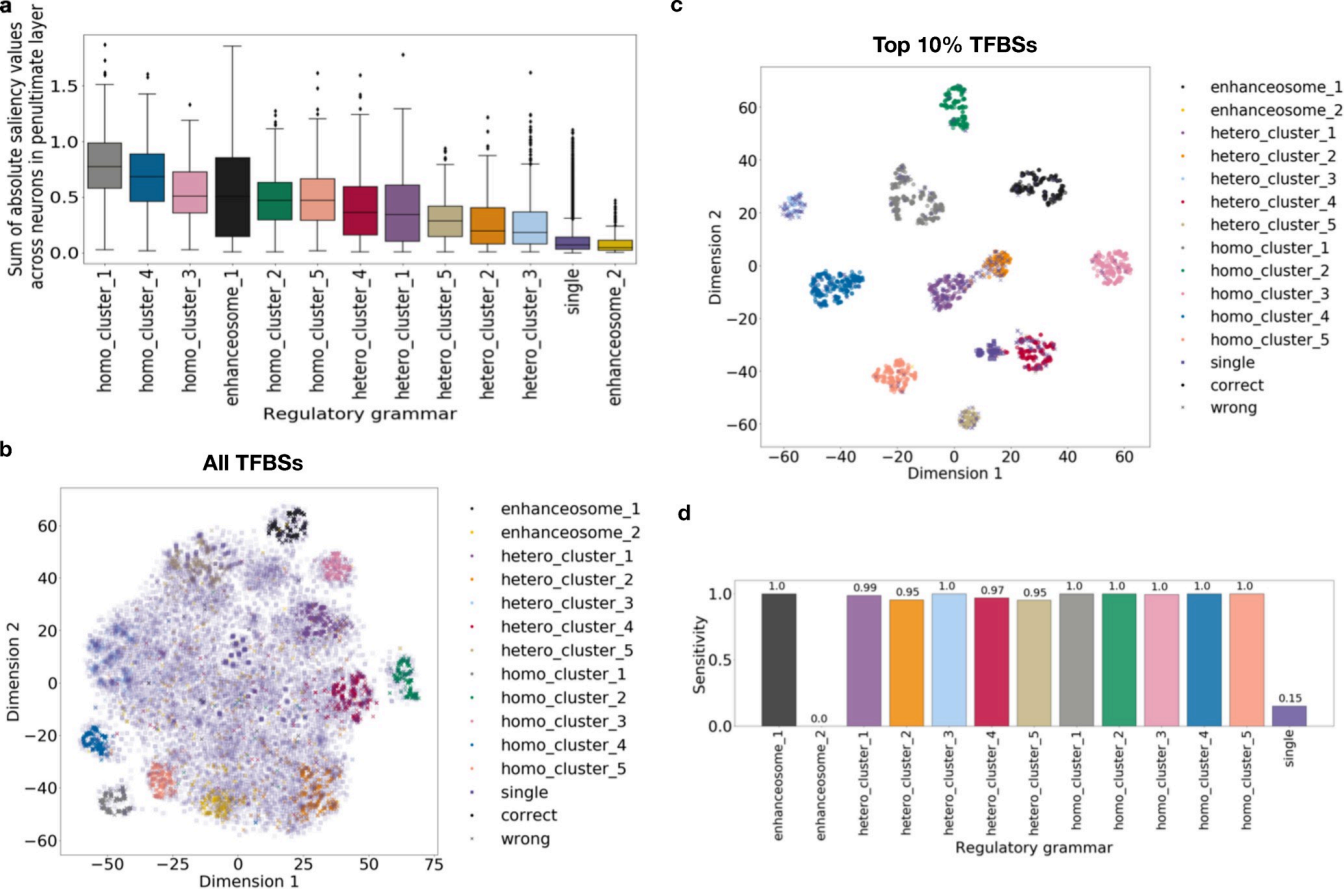
Regulatory grammar can be learned by ResNet when TFBSs are outside of regulatory grammars and there is heterogeneity in the regulatory sequence categories. (a) Sum of saliency values for TFBSs in each regulatory grammar across neurons in penultimate layer. (b) Actual labels of simulated regulatory grammar of the TFBS overlaid on t-SNE visualization of TFBS saliency values across neurons. (c) Actual labels of simulated regulatory grammar of the TFBS filtered to only those in the top 10% sum of saliency values across neurons in penultimate layer overlaid on the t-SNE visualization. (d) The sensitivity of predicted labels in (c) across regulatory grammars.

We trained a ResNet model on this 80% non-grammar TFBSs dataset with the five heterogenous classes as output categories against 8-mer shuffled negatives. We found that the TFBSs outside of the regulatory grammars (single TFBS) have lower saliency values compared to the TFs in simulated regulatory grammars (Fig 5A) except for those in enhanceosome 2.

Next, we performed unsupervised clustering analysis as in the previous sections (Fig 5B). Although the TFBSs in regulatory grammars still cluster, many of the TFBSs outside of regulatory grammar overlap the TFBSs in regulatory grammars in t-SNE space. This makes identifying the regulatory grammars challenging. To better reconstruct the regulatory grammar from the unsupervised clustering analysis, we took advantage of the fact that the non-grammar TFBSs have lower saliency values and only kept the TFBSs with top 10% sum of saliency values across neurons in the penultimate layer. Intuitively, this filtering helps improve the reconstruction of regulatory grammar by only focusing on TFBSs with high influence on the prediction. We repeated the unsupervised clustering analysis on these filtered TFBSs (Fig 5C). We found that nearly all TFBSs outside of regulatory grammars are filtered out (97.7%) and a smaller fraction of TFBSs in regulatory grammars are filtered (59.3%). After filtering, the remaining TFBSs are sufficient to reconstruct 11 of the 12 simulated regulatory grammars. The regulatory grammar that we failed to reconstruct, enhanceosome 2, has the lowest sum of saliency values across neurons in the penultimate layer (Fig 5A), suggesting that is was not important to learn this grammar to obtain accurate predictions. The neural network may achieve accurate predictions through elimination and therefore did not need to learn all 12 regulatory grammars.

These results suggest that even with only a small fraction of TFBSs in regulatory grammars and heterogeneity in the output categories, we can still reconstruct most of the simulated regulatory grammars.

### Regulatory grammar cannot be learned if multiple grammars are able to distinguish one regulatory sequence class from another

As shown in Figs 4 and 5, some regulatory grammars, especially enhanceosome 2, are reconstructed from ResNet model with limited accuracy. This suggests that the “essentiality” of a regulatory grammar may influence the ability to reconstruct regulatory grammars from the model. In other words, if a neural network can make accurate predictions without learning certain regulatory grammars, then these non-essential regulatory grammars may not be learned during training and therefore cannot be reconstructed from the resulting model. To further investigate this hypothesis, we simulated three heterogenous regulatory classes (Table 1) with non-overlapping subsets of regulatory grammars, so that multiple regulatory grammars could distinguish one heterogenous regulatory class from another. Then we trained the model against TF-shuffled negative sequences. By setting up the training this way, the model has to distinguish sequences with TFBSs in regulatory grammars from those with TFBSs not in regulatory grammars. However, the model does not need to learn all the regulatory grammars or distinguish one regulatory grammar from the other to make accurate predictions.

**Table 1:** Simulated heterogenous regulatory sequence classes with multiple regulatory grammars that can distinguish one class from another.

	Regulatory grammars in sequence type 1	Regulatory grammars sequence type 2
Heterogeneous Regulatory Sequence Class 1	homotypic cluster 1, homotypic cluster 2	homotypic cluster 4, heterotypic cluster 4
Heterogeneous Regulatory Sequence Class 2	heterotypic cluster 1, heterotypic cluster 2	homotypic cluster 5, enhanceosome 1
Heterogeneous Regulatory Sequence Class 3	homotypic cluster 3, heterotypic cluster 3	heterotypic cluster 5, enhanceosome 2

**Fig 6:**
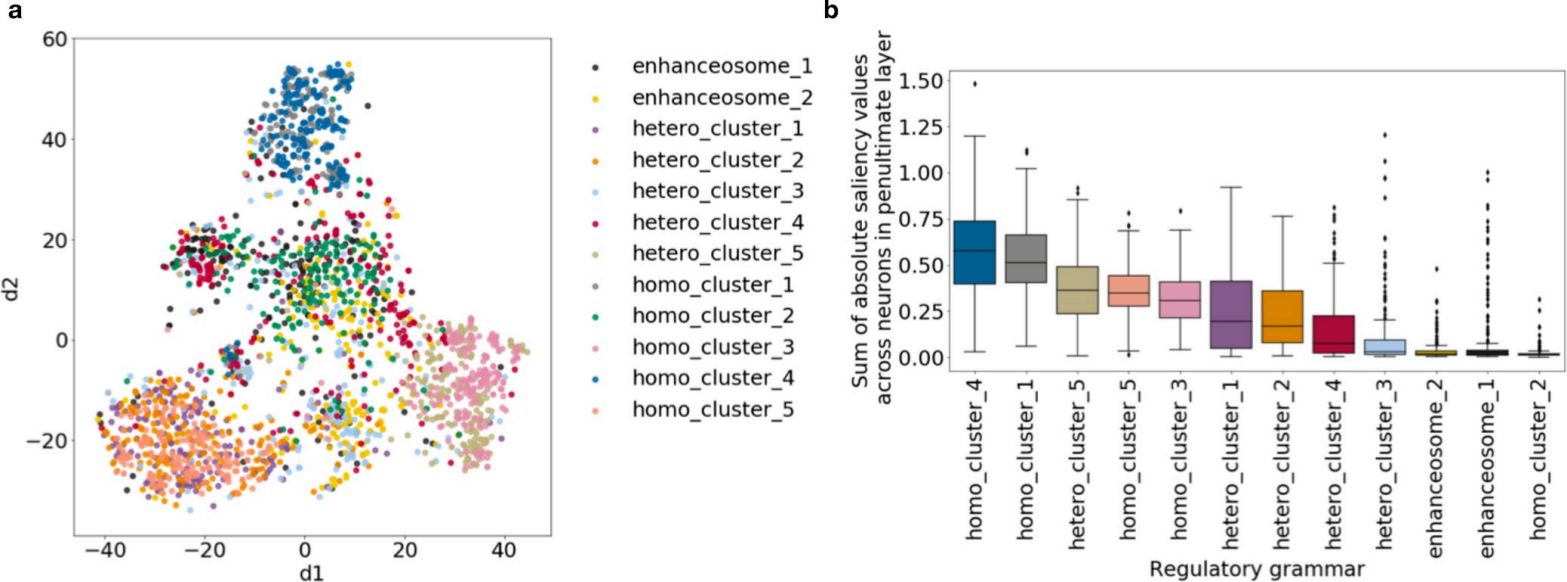
The ResNet model fails to learn the correct representation of individual grammars when there are multiple regulatory grammars that can distinguish one heterogenous regulatory class from another. For this simulation, we created three heterogeneous regulatory sequence classes with no overlap among their grammars (Table 1) and applied our interpretation approach. a) Actual labels of simulated regulatory grammars of the TF binding sites overlaid on t-SNE visualization of TFBS saliency values across neurons. The TFBSs do not separate according to their grammars. b) Sum of saliency values for TFBSs in each regulatory grammar across neurons in the penultimate layer.

As expected, the model performed well at distinguishing positives and negatives (average auROC 0.995, auPR 0.978). However, when visualizing the saliency values of TFBSs of the neurons in the penultimate layer, there is limited resolution to recover individual regulatory grammars; multiple regulatory grammars have similar saliency profiles and overlap in the t-SNE space (Fig 6A). This observation is consistent with our hypothesis that if there are multiple regulatory grammars that can distinguish one class of sequences from another, the neural network will not learn to distinguish one regulatory grammar from another nor learn all the distinct regulatory grammars.

For example, in Table 1, for *Heterogeneous Regulatory Sequence Class 1*, there are two types of simulated enhancer sequences. Sequences in the first type have homotypic cluster 1 as well as homotypic cluster 2. Because neither of these grammars are used in any other *Heterogeneous Regulatory Sequence Classes*, the model would only need to learn one of homotypic cluster 1 and homotypic cluster 2 to tell that a sequence belongs to *Heterogeneous Regulatory Sequence Class 1*. Fig 6B suggests that in our simulation, the model learned homotypic cluster 1. The same logic can be applied to the second type of simulated sequence in *Heterogeneous Regulatory Sequence Class 1*. The model only needs to learn either homotypic cluster 4 or heterotypic cluster 4. In our simulation, the model learned homotypic cluster 4 (Fig 6B). Moreover, the model does not need to distinguish homotypic cluster 1 (in the first type sequences) from homotypic cluster 4 (in the second type sequences). This is why we see TFBSs in homotypic cluster 1 (gray) are clustered with homotypic cluster 4 (blue) in Fig 6A. Applying the same logic to *Heterogeneous Regulatory Sequence Class 3* explains why homotypic cluster 3 and heterotypic cluster 5 have higher importance than the other two grammars and are clustered together (Fig 6B). Similarly, for *Heterogeneous Regulatory Sequence Class 2*, homotypic cluster 5 and heterotypic cluster 1 and 2 have higher importance than the other two grammars and are clustered together.

This scenario is likely in many real enhancer classification tasks, especially when sequences in one class are distinctly different from another with multiple predictive sequence patterns. This would make reconstruction of full individual regulatory grammars challenging.

### ResNet trained on mouse developmental enhancers identifies a known heart heterotypic TF cluster

**Fig 7:**
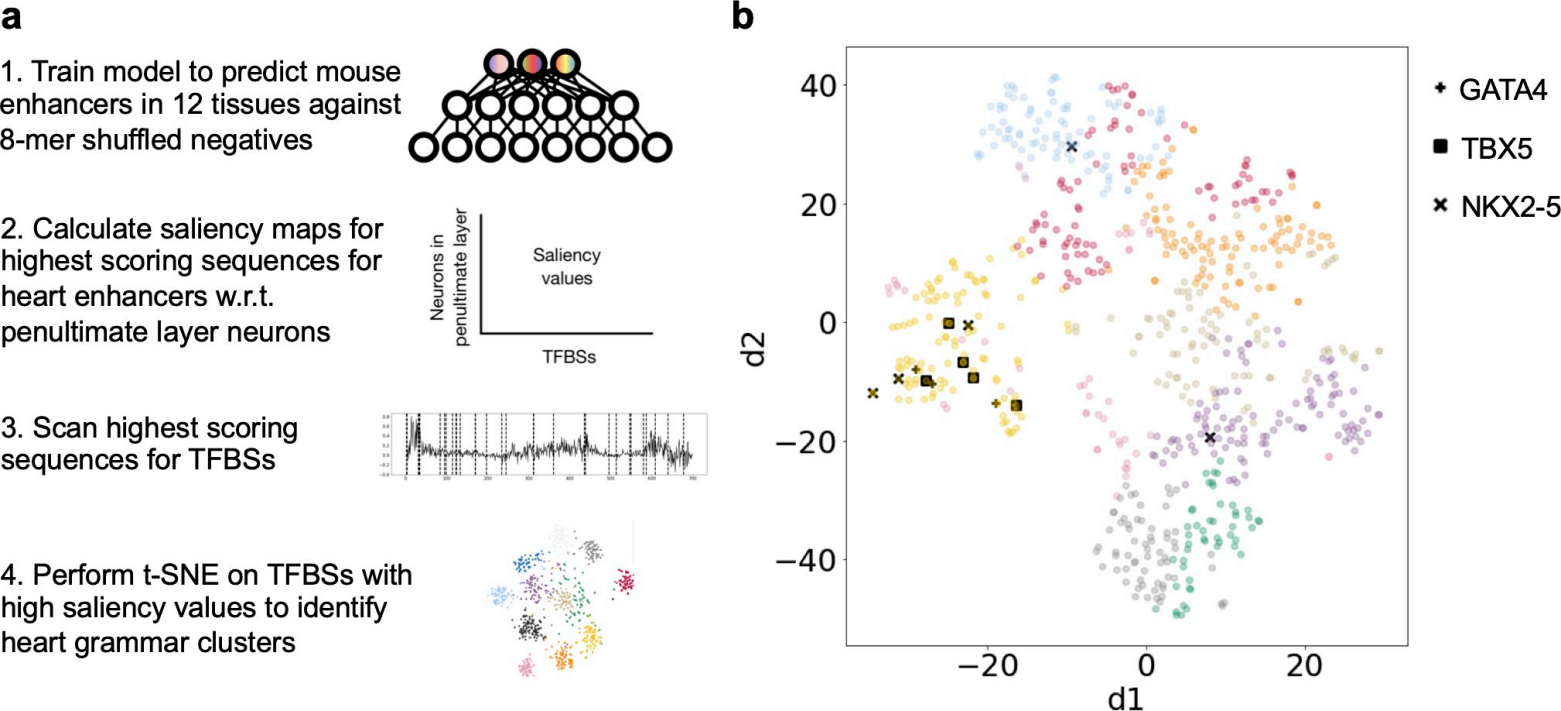
ResNets identify known heart heterotypic cluster when trained on mouse enhancers. We trained a ResNet on developmental mouse enhancers from 12 tissues identified from histone modifications (S6 Fig) and applied our saliency map approach to interpret the trained network. a) Pipeline for identifying regulatory grammar in mouse developmental heart enhancers. b) t-SNE visualization of clustered TFBS saliency maps from top scoring heart enhancer sequences. Clusters determined by k-means with k = 9 are indicated by color (S6C Fig). Instances of NKX2-5, TBX5, and GATA4 motifs are labelled with shapes. These factors form an essential heterotypic cluster during heart development and are significantly enriched in cluster 4 (S5 Table).

The lack of well-characterized regulatory grammars required us to develop and evaluate our methods on simulated data. However, as a preliminary test of our approaches for regulatory grammar identification on real enhancers, we applied our approach to enhancers from mouse development (Fig 7A). We sought to test whether we could identify the transcription factors TBX5, NKX2-5, and GATA4, which are known to function as a heterotypic cluster to coordinately control gene expression during cardiac differentiation [64].

First, we trained a ResNet on mouse E14.5 developmental enhancers from 12 tissues, including heart, limb, neural tube, kidney, embryonic facial prominence, liver, intestine, lung, stomach, forebrain, midbrain, and hindbrain [66], against 8-mer shuffled negatives (Methods). The model achieved moderate accuracy for the different tissues (auROC 0.71–0.81, auPR 0.1–0.38, S6 Fig). The moderate accuracy is partially due to the use of 8-mer shuffled sequences to create a difficult negative set rather than a simpler negative set, since our goal was to encourage the neural network to learn grammars rather than individual transcription factor motifs.

Next, we calculated saliency maps for the same set of sequences using the 8-mer shuffled negative sequences as reference with respect to the neurons in the penultimate layer. We then applied FIMO to identify TFBSs in the highest scoring 100 sequences for the heart enhancers. We visualized with t-SNE the saliency profile of TBX5, NKX2-5, GATA4, and representative TFs from each TF family (based on median motif counts within each TF family). To simplify visualization and remove TFBS unlikely to be in a grammar, we focused on the TFBSs with the top 30% sum of saliency values. This threshold removed low importance TFBSs, but kept enough for the clustering analysis. Then, we applied k-means clustering (k = 2–12) to the saliency map matrix (Fig 7B); k = 9 yielded the best silhouette score (S6C Fig). We then tested for TFBSs enrichment in the clusters.

The motifs for seven TFs are significantly enriched in cluster 4 (S5 Table), including the heart heterotypic cluster TFs (TBX5, NKX2-5, and GATA4). All five TBX5 binding sites (p = 9.1e-5, Fisher’s exact test), all three GATA4 binding sites (p = 0.00037), and three out of five NKX2-5 binding sites (p = 0.029) are in cluster 4 (Fig 7B). This suggests that that ResNet learned the heterotypic cluster. Furthermore, three of the additional TFBSs enriched in cluster 4—BRAC/TBXT (p = 0.00018), GATA3 (p = 0.024), TBX21 (p = 0.038)—have binding motifs very similar to members of the heterotypic cluster. GATA3 is the representative of the GATA zinc-finger family that GATA4 belongs to, and the TBX21 and TBXT motifs are similar to TBX5. This suggests that their enrichment reflects their similarity to components of the regulatory grammar. The final enriched motif, NR6A1 (p = 0.0033), has high similarity to retinoid X receptor (RXR) motifs, and it is not clear if it is related to the heterotypic cluster.

These preliminary results demonstrate that our saliency map approach identifies the components of a known regulatory grammar from deep neural networks trained on a complex real enhancer dataset. However, further work is needed to enable comprehensive and reliable discovery and validation of novel grammars.

## Discussion

We trained a variant of DNNs, ResNets, to model sequences with simulated regulatory grammars (combinatorial binding of TFs). Then we developed a gradient-based unsupervised clustering approach to interpret the features learned by neurons in the intermediate layers of the neural network. We found that ResNets can model the simulated regulatory grammars even when there is heterogeneity in the regulatory sequences and a large fraction of TFBSs outside of regulatory grammars. Finally, we trained a ResNet on mouse developmental enhancers and were able to identify components of a known heterotypic cluster of TFs active in heart development.

We also identified scenarios when the ResNet model failed to learn the regulatory grammar. The networks strive to learn simple representations of the training data. As a result, the ResNet models in our studies failed to learn the simulated regulatory grammar when there is a lack of constraint in negative training samples or between the positive output categories. For instance, we found that the choice of negative training samples influences the ability of the neural network to learn regulatory grammar. The model trained against no negatives, short k-mer shuffled negatives (k = 1, 2, 4, 6), or very long k-mer shuffled negatives (k = 12) did not learn accurate representations of simulated regulatory grammars and often misclassified TF-shuffled negatives as positives. The model trained against 8-mer shuffled negatives performed the best when evaluated on the TF-shuffled negatives. This is because when shorter k-mers (k< = 6) are used to generate the negative training samples, the neural network can distinguish the positives from negatives by learning the individual TF motifs, many of which are longer than 6 bp, rather than learning the regulatory grammar of the TFs. With longer k-mers (k = 12), the reason is likely that k-mers are not well shuffled in the negatives and very similar to the positives. Indeed, the ResNet model trained against 12-mer shuffled negatives has lower accuracy (auPR 0.506). The 8-mer shuffled negative provides a sweet spot where the negatives are well shuffled and the network is forced to learn the TF motifs and regulatory grammars. Another challenging situation occurs when there are multiple sequence features that can distinguish one output category from another. Under this scenario, it is not necessary for the neural network to accurately learn all the features nor distinguish one feature from another.

In addition to these scenarios, there is also another situation in which the ResNet model failed to learn the regulatory grammar. When the majority of the TFBSs are not in a regulatory grammar, the non-grammar TFBSs overlap those in regulatory grammars in the unsupervised clustering analysis and make it impossible to recover the grammars. Fortunately, we could use the observation that many of the TFBSs outside of regulatory grammars have low saliency values to filter out those TFBSs, and focus the unsupervised clustering analysis on TFBS with high saliency values to improve the accuracy of grammar reconstruction. This gradient magnitude- based filtering method may be less efficient when there is an overwhelmingly large number of TFBSs outside of regulatory grammar and larger sample sizes might be needed to train the neural network to better retrieve the regulatory grammars.

Large-scale evaluation of our approach on real data is not possible due to the small number of known regulatory grammars. However, to begin to explore the performance of our approach on real enhancer sequences, we demonstrated that it highlights members of a known heterotypic cluster of three TFs (TBX5, GATA4, and NKX2-5) essential to mouse heart development. This preliminary of analysis of mouse developmental enhancers is intended as a proof of principle of the potential utility of our approach in identifying candidate regulatory grammars. In the future, the same approach can be applied to enhancers in other cellular contexts on a larger scale, but in the absence of additional known grammars, substantial work will be needed to reconstruct and validate proposed grammars. We anticipate that this will require integration of machine learning methods with high-throughput experimental strategies for evaluating gene regulatory activity of DNA sequences with different binding site combinations.

While we demonstrate potential to interpret biologically relevant patterns learned by deep neural network models in some realistic scenarios, our work has several caveats. First, the synthetic dataset and proposed methods assume that combinatorial binding of TFs does not change their motifs. However, this assumption is not always true. In vitro analyses of the combinatorial binding of pairs of TFs indicate that many pairs of TFs have different binding motifs when they bind together compared to their consensus motifs [17]. Although there is nothing preventing the neural network from learning such altered motifs, the unsupervised clustering methods based on individual TFBS may have limited accuracy in identifying such altered motifs. Second, we did not simulate noisy labels in the synthetic dataset which could occur in the real regulatory sequence prediction tasks. The common methods of experimentally finding enhancers, such as ChIP-seq on histone modifications, DNase-Seq, CAGE-seq, and MPRAs, often produce mislabeled regulatory regions and vague region boundaries. This could be improved in the future by integrating methods for learning from noisy labeled data.

In summary, we demonstrated the power and limitations of deep convolutional neural networks at modeling regulatory grammars and provided a backpropagation gradient based unsupervised learning approach to retrieve and interpret the patterns learned by inner layers of the neural network. Our work indicates that DNNs can learn biologically relevant TFBS combinations in certain settings with carefully defined training data; however, in many common scenarios, we should be cautious when interpreting the biological implications of features learned by DNA-sequence-based DNNs. We anticipate that biologically informative machine-learning-based interpretation of regulatory sequences can be further improved with better annotated, less noisy training data and more sophisticated models.

## Methods

### Simulated sequence generation and analysis

#### Simulation of regulatory grammar

We used TF binding motifs from the HOCOMOCO v11 database [67]. To make sure that the TF motifs are distinct and diverse, we select one TF from each TF subfamily. This results in a set of 26 TFs (S1 Table). Then the selected TFs are arranged into three types of regulatory grammar representing homotypic clusters, heterotypic clusters, and enhanceosomes.

For the homotypic cluster, we simulated multiple non-overlapping occurrences (3–5) of the same TF in a small window (120 bp) at random locations. For the heterotypic clusters, we simulated a set of four diverse TFs in a small window (120 bp) at random non-overlapping locations. Each TF occurs once in the heterotypic cluster. For the enhanceosome, we simulated a set of four TFs in a small window with fixed order and spacing. Because it is possible in real enhancers that the same TF factor is used in different regulatory grammars, we allow some of TFs to occur in more than one grammar. We simulated five homotypic TF clusters, five heterotypic clusters and two enhanceosomes (S2 Table).

#### Simulation of regulatory sequences with different regulatory grammars

To mimic common enhancer prediction tasks, such as predicting enhancers from different cellular contexts, we designed twelve regulatory sequence classes (S3 Table) with each regulatory sequence class representing one type of enhancer (e.g., enhancers active in a given context). Sequences in each class have two different regulatory grammars. Because it is possible that the same regulatory grammar is used in regulatory sequences in different cellular contexts, we allow one regulatory grammar occur in two different regulatory sequence classes. For instance, the first regulatory sequence class has homotypic cluster 1 and heterotypic cluster 1, then the second regulatory sequence class has heterotypic cluster 1 and homotypic cluster 2 and then the third regulatory sequence class has homotypic cluster 2 and heterotypic cluster 3, etc. Next, we randomly generated background DNA sequences of 3000 bp based on equal probability of A, G, C, T and inserted 2–4 of each simulated regulatory grammar at random locations into these background sequences based on the corresponding regulatory class.

#### Multiclass classification and heterogenous class classification

We performed two types of classification: i) multiclass classification in which each output neuron represents a homogenous set of regulatory sequences and ii) heterogenous class classification in which each output neuron represents a heterogenous set of regulatory sequences. The heterogenous class classification task assumes that in the real enhancer prediction tasks, enhancers in one category (e.g., specific cellular context) may have a heterogenous set of sequences harboring different sets of regulatory grammars.

The multiclass classification task has twelve homogeneous output classes, each one corresponding to sequences representing one regulatory sequence class. The heterogenous class classification (S4 Table) has five heterogeneous output classes, each one corresponding to a subset of regulatory sequence classes. More specifically, heterogeneous class 1 has regulatory sequence class 1, 3, and 5; heterogeneous class 2 has regulatory sequence class 2, 4, and 6; heterogeneous class 3 has regulatory sequence class 7, 9, and 11; heterogeneous class 4 has regulatory sequence class 5, 8, and 10; heterogeneous class 2 has regulatory sequence class 1, 6, and 12.

#### Negative sequences

We used three approaches to generate negatives when training the classifiers: no negatives, k-mer shuffled negatives, and TF-shuffled negatives. For the k-mer shuffled negative sequence set, we matched the frequency of k-mers (k = 1, 2, 4, 8, 12) in the negatives to the simulated regulatory (positive) sequences. For the TF-shuffled sequence set, we randomized the positions of TFBS in the simulated regulatory sequences to break the membership of TFs in regulatory grammars.

#### Model design and training

DNNs have achieved the state-of-art performance on regulatory sequence prediction [45, 46]. The integration of a convolution operation into standard neural networks enables learning common patterns that occur at different spatial positions, such as TF motifs in the DNA sequences. Here we use a residual deep convolutional neural networks (ResNets), a variant of DNNs that allows connections between non-sequential layers [68] to model the regulatory sequences. Each simulated DNA sequence is one-hot-encoded, which is represented by a sequence length x 4 matrix with columns representing A, G, C and T.

The basic layers in the network include a convolutional layer, batch normalization layer, pooling layer, and fully connected layer. Every two convolutional layers are grouped into a residual block where an identity shortcut connection adds the input to the residual block to the output of the residual block. This additional identity mapping is an efficient way to deal with vanished gradients that occur in neural networks with large depth and improves performance in many scenarios. The batch normalization layers are added after the activation of each residual block. Batch normalization [69] helps reduce the covariance shift of the hidden unit and allows each layer of a neural network to learn more independently of other layers. The pooling layers are added after each batch normalization layer. Finally, a dense (fully connected) layer and an output layer are added at the top of the neural network. We used 4 residual blocks, each has two convolutional layers with 32 neurons. The final residual block is connected to a dense layer with 32 neurons and then connected to output layer (S1 Fig). We found the above neural network structure (ResNet) performed well in all of our simulation tasks while a 3-layer convolutional neural network with alternating convolutional layers and maxpooling layers cannot, suggesting the benefit of using a much deeper neural network at modeling enhancer regulatory grammar.

We used rectified (ReLU) activation for all the residual blocks and sigmoid activation for the output fully connected layer activation. We used binary cross-entropy as the loss function and Adam [70] as the optimizer. We implemented the model using the keras library with TensorFlow as the backend [71].

### Model interpretation and grammar reconstruction

#### Computing saliency values with respect to neurons

We considered two gradient calculating approaches for estimating the importance of each nucleotide in the input sequence with respect to each neuron’s activation. The first is guided back-propagation in which we calculated the gradient of the neuron of interest with respect to the input through guided back-propagation and then multiplied the gradient by input sequences. The second is calculating the DeepLIFT score [56] of the neuron of interest with respect to the input using the DeepLIFT algorithm implemented in SHAP [72] against the TF-shuffled negatives and then multiplying the DeepLIFT score by input sequences. We refer the resulting values from the above as saliency values and the vector of saliency values for an input sequence as saliency map. We found that the saliency maps calculated using DeepLIFT approach performed the better than guided back-propagation (S2 Fig). Therefore, for all the main text results we present were calculated with the DeepLIFT approach.

#### Analysis of TF saliency maps

To analyze which TFs are learned by a specific neuron, we calculate the gradient of a TF binding site with respect to a neuron by averaging a 10 bp window from the start position of the TF binding site. Then, we visualize the distribution of saliency values of the binding sites of each TF in a specific regulatory grammar with respect to a neuron with box plot.

The median saliency values of the binding sites of each TF in a specific regulatory grammar with respect to neurons is stored in a matrix with the shape of number of neurons by the number of TFs. This data matrix is first scaled by column to identify which neurons mostly detect the TF and the scaled matrix is used to generate a heatmap. Then, we performed hierarchical clustering with k = 12 (12 is the number of simulated regulatory grammars) or k = 13 (when there are non-grammar TFBSs) for both neurons and TFs based on the same data matrix.

#### t-SNE and k-means clustering of TFBS

To reconstruct the regulatory grammar and evaluate how accurately neurons in a layer capture the simulated regulatory grammar, we performed a two-dimensional t-SNE and a k-means clustering (k = 12) of TFBS using their saliency value profiles across neurons in a layer. To assign the name of regulatory grammar of a predicted cluster, we used a majority vote, which is the majority of the true labels of regulatory grammar in that cluster. We visualize the k-means clustering by overlaying the predicted regulatory grammar from k-means clustering on top of the t-SNE visualization. We evaluated the performance at reconstructing the regulatory grammar by two metrics: the accuracy ((TP+TN)/All) and the sensitivity (TP/(TP+FN)) of the regulatory grammar predictions.

### Mouse developmental enhancer analysis

#### Mouse developmental enhancers and 8-mer shuffled negatives

We analyzed H3K27ac and H3K4me1 peak files from mouse heart, limb, neural tube, kidney, embryonic facial prominence, liver, intestine, lung, stomach, forebrain, midbrain, hindbrain at E14.5 [66]. We defined enhancers as regions with the H3K27ac mark without the H3K4me1 mark. Then, we partitioned mouse genome into 200 bp bins and annotated each bin as an enhancer in a tissue if more than 50% if its base pairs overlap with an enhancer in that tissue. We kept all 634,087 bins that are active enhancers in at least one tissue. Enhancers may be longer than 200 bp and including flanking regions often improves enhancer model accuracy [45]; thus, we added 250 bp flanking regions at each side of the 200 bp regions to create 700 bp regions. We then generated the same number of 8-mer shuffled negatives as enhancer regions using fasta-shuffle-letters from the MEME suite. Finally, we one-hot encoded the enhancer regions and 8-mer shuffled negatives. This results in an input matrix of (1268174, 700, 4) and an output matrix of (1268174, 12).

#### Model design and training

We used a similar residual neural network as those used for the simulated data. However, given the larger size of the training data, we added an additional residual block, used more neurons in each residual block convolutional layer (128, 256, 256, 512, 512), and used 1024 in the penultimate fully connected layer.

## Supporting information

S1 Fig The structure of the ResNet model. (PNG)

S2 Fig The DeepLIFT score is better at reconstructing the regulatory grammar compared to guided back-propagation gradient. a. True and predicted labels of simulated regulatory grammar of the TF binding sites overlaid on t-SNE visualization. b. The sensitivity (TP/TP+FN) of predicted labels of regulatory grammar using DeepLIFT score x Input or Guided back-propagation gradient.
(PDF)

S3 Fig The neural network learned individual TF binding motifs in the lower convolutional layer and gradually build up its understanding of regulatory grammar in higher level layers. a. Simulated TF motifs are learned by neurons in the third convolutional layer. From left to right are four selected examples, neuron 9 learned the FOS motif; neuron 20 learned the COT2 motif; neuron 22 learned the P53 motif; neuron 25 learned ERR1 motif. b. From layer 7 (third convolutional layer) to Layer 43 (the penultimate dense layer), the ResNet model gradually learned the regulatory grammar. The correlation matrix of the saliency value profiles of TFs in a specific regulatory grammar is plotted as the heatmap. In layer 7, TFs from the same regulatory grammar are not clustered. In layer 37, TFs within the same regulatory grammar begin to have a higher correlation. In layer 43, TFs within the same regulatory grammar have near perfect correlation.
(PNG)

S4 Fig ResNet model trained on simulated regulatory sequences and 8-mer shuffled negatives. Heatmap of the median gradient of the binding sites of each TF in a specific regulatory grammar (x axis) across neurons of the penultimate layer (y axis). The order of x and y axis labels are determined by hierarchical clustering shown on side. The color bars indicate the group label assigned by hierarchical clustering.
(PNG)

S5 Fig The accuracy of ResNet model trained for heterogeneous multilabel classification with no negatives or against k-mer shuffled negatives. (PNG)

S6 Fig The accuracy of ResNet model trained on mouse developmental enhancers from 12 tissues. a. ROC curve. b. PR curve. c. The silhouette score of k-means clustering with k from 3 to 10.
(PNG)

S1 Table Transcription factor used in constructing regulatory grammars. (PDF)

S2 Table Simulated regulatory grammar. (PDF)

S3 Table Simulated regulatory classes. (PDF)

S4 Table Simulated heterogenous regulatory classes. (PDF)

S5 Table Enrichment of TF binding sites in cluster 4. (CSV)
