# Supplementary material for: Learning and interpreting the gene regulatory grammar in a deep learning framework
Source: PLoS Comput Biol. 2020 Nov 2;16(11):e1008334. doi: 10.1371/journal.pcbi.1008334 (PMC7660921; doi:10.1371/journal.pcbi.1008334)
Supplement: S1 Table — (PDF) [file pcbi.1008334.s007.pdf]

Table 1: Transcription factor used in constructing regulatory grammars

TF	logo	regulatory rammar
NFYA	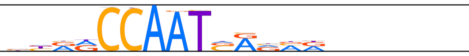	homo_cluster_1
POU3F2	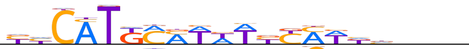	homo_cluster_2
TP53	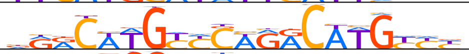	homo_cluster_3
ESRRA	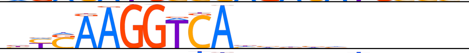	homo_cluster_4
IRF1	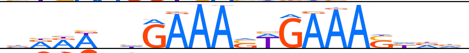	homo_cluster_5
KLF1	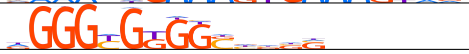	hetero_cluster_1
MEF2A	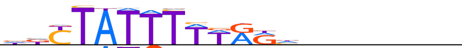	hetero_cluster_1
YY1	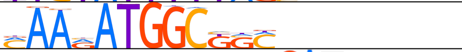	hetero_cluster_1, hetero_cluster_2
GATA1	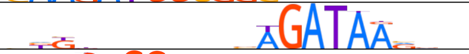	hetero_cluster_1, enhanceosome_2
RUNX3	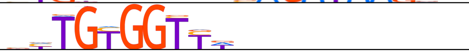	hetero_cluster_2
PDX1	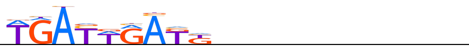	hetero_cluster_2
BCL11A	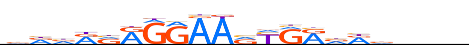	hetero_cluster_2, hetero_cluster_3
E2F1	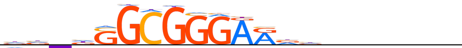	hetero_cluster_3
FOXA1	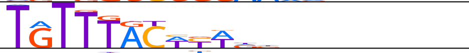	hetero_cluster_3
NR2F2	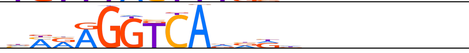	hetero_cluster_3, hetero_cluster_4
VDR	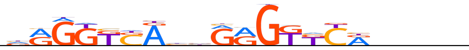	hetero_cluster_4
BACH1	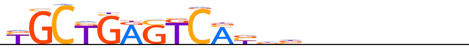	hetero_cluster_4
FOS	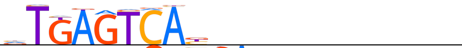	hetero_cluster_4, hetero_cluster_5
MAFG	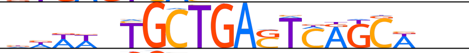	hetero_cluster_5
ERG	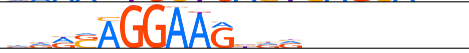	hetero_cluster_5
STAT5B	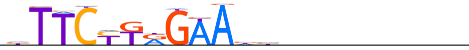	hetero_cluster_5, enhanceosome_1
TCF7	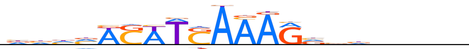	enhanceosome_1
MAX	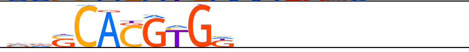	enhanceosome_1
LYL1	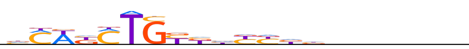	enhanceosome_1, enhanceosome_2
PBX1	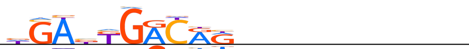	enhanceosome_2
CEBPA	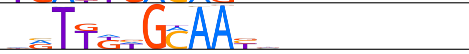	enhanceosome_2
