# Supplementary material for: Learning and interpreting the gene regulatory grammar in a deep learning framework
Source: PLoS Comput Biol. 2020 Nov 2;16(11):e1008334. doi: 10.1371/journal.pcbi.1008334 (PMC7660921; doi:10.1371/journal.pcbi.1008334)
Supplement: S2 Table — (PDF) [file pcbi.1008334.s008.pdf]

Table 2: Simulated regulatory grammar

type	name	TFs
homotypic cluster	homo_cluster_1	NFYA
homotypic cluster	homo_cluster_2	POU3F2
homotypic cluster	homo_cluster_3	TP53
homotypic cluster	homo_cluster_4	ESRRA
homotypic cluster	homo_cluster_5	IRF1
heterotypic cluster	hetero_cluster_1	KLF1, MEF2A, YY1, GATA1
heterotypic cluster	hetero_cluster_2	RUNX3, PDX1, BCL11A, YY1
heterotypic cluster	hetero_cluster_3	E2F1, FOXA, NR2F2, BCL11A
heterotypic cluster	hetero_cluster_4	VDR, BACH1, FOS, NR2F2
heterotypic cluster	hetero_cluster_5	MAFG, ERG, STAT5B, FOS
enhanceosome	enhanceosome_1	TCF7, MAX, LYL1, STAT5B
enhanceosome	enhanceosome_2	PBX1, CEBPA, GATA1, LYL1
