# Supplementary material for: Learning and interpreting the gene regulatory grammar in a deep learning framework
Source: PLoS Comput Biol. 2020 Nov 2;16(11):e1008334. doi: 10.1371/journal.pcbi.1008334 (PMC7660921; doi:10.1371/journal.pcbi.1008334)
Supplement: S3 Table — (PDF) [file pcbi.1008334.s009.pdf]

Table 3: Simulated regulatory classes

| regulatory sequence class | regulatory grammars |
|---|---|
| regulatory_class1 | homo_cluster_1, hetero_cluster_1 |
| regulatory_class2 | hetero_cluster_1, homo_cluster_2 |
| regulatory_class3 | homo_cluster_2, hetero_cluster_2 |
| regulatory_class4 | hetero_cluster_2, homo_cluster_3 |
| regulatory_class5 | homo_cluster_3, hetero_cluster_3 |
| regulatory_class6 | hetero_cluster_3, homo_cluster_4 |
| regulatory_class7 | homo_cluster_4, hetero_cluster_4 |
| regulatory_class8 | hetero_cluster_4, homo_cluster_5 |
| regulatory_class9 | homo_cluster_5, hetero_cluster_4 |
| regulatory_class10 | hetero_cluster_5, enhanceosome_1 |
| regulatory_class11 | enhanceosom_1, enhanceosome_2 |
| regulatory_class12 | enhanceosom_2, homo_cluster_1 |
