# Supplementary material for: Learning and interpreting the gene regulatory grammar in a deep learning framework
Source: PLoS Comput Biol. 2020 Nov 2;16(11):e1008334. doi: 10.1371/journal.pcbi.1008334 (PMC7660921; doi:10.1371/journal.pcbi.1008334)
Supplement: S4 Table — (PDF) [file pcbi.1008334.s010.pdf]

Table 4: Simulated heterogenous regulatory classes

heterogenous regulatory sequence class	regulatory classes
heterogenous_regulatory_class1	regulatory_class1, regulatory_class3, regulatory_class5
heterogenous_regulatory_class2	regulatory_class2, regulatory_class4, regulatory_class6
heterogenous_regulatory_class3	regulatory_class7, regulatory_class9, regulatory_class11
heterogenous_regulatory_class4	regulatory_class5, regulatory_class8, regulatory_class10
heterogenous_regulatory_class5	regulatory_class1, regulatory_class6, regulatory_class12
